# Tetracycline, an Appropriate Reagent for Measuring Bone-Formation Activity in the Murine Model of the *Streptococcus mutans*-Induced Bone Loss

**DOI:** 10.3389/fcimb.2021.714366

**Published:** 2021-09-13

**Authors:** Yuna Hirohashi, Shingo Kamijo, Masud Khan, Masaomi Ikeda, Meiko Oki, Khairul Matin, Fatma Rashed, Kazuhiro Aoki

**Affiliations:** ^1^Department of Basic Oral Health Engineering, Graduate School of Medical and Dental Sciences, Tokyo Medical and Dental University, Tokyo, Japan; ^2^Department of Oral Prosthetic Engineering, Graduate School of Medical and Dental Sciences, Tokyo Medical and Dental University, Tokyo, Japan; ^3^Department of Cariology and Operative Dentistry, Graduate School of Medical and Dental Sciences, Tokyo Medical and Dental University, Tokyo, Japan; ^4^Endowed Department of International Oral Health Science, Tsurumi University School of Dental Medicine, Tsurumi, Yokohama, Japan; ^5^Department of Oral Biology, Faculty of Dentistry, Damanhour University, El Behera, Egypt

**Keywords:** tetracycline, bone formation indices, fluorescent labeling, *Streptococcus*. *mutans*, bone loss

## Abstract

Tetracycline is used as a fluorescent reagent to measure bone formation activity in bone histomorphometric analyses. However, there is a possibility to lead a different conclusion when it is used in a bacteria-infected murine model since the tetracycline is considered to work as an antibiotic reagent. There are non-antibiotic fluorescent reagents such as alizarin and calcein for measuring bone formation activity. The purpose of this study was to clarify whether tetracycline could be an appropriate reagent to measure bone formation activity in a murine bacterial model in the same way as a non-antibiotic fluorescent reagent. We used *Streptococcus mutans* (*S. mutans*), a normal inhabitant in the oral cavity and tetracycline-sensitive bacteria, for inducing the bacterial model. The murine bacterial model was generated by intravenously inoculating *S. mutans* to the tail vein, followed immediately by the injection of the first fluorescent reagent, and the second one was injected 2 days prior to euthanization. After one day of inoculation with *S. mutans*, the subcutaneously injected alizarin had a similar colony count derived from the liver and the bone marrow tissue compared to the phosphate buffered saline (PBS)-injected control group. On the other hand, subcutaneous injection of tetracycline led to a significantly lower colony count from the liver compared to alizarin- or calcein-injected group. However, on day seven, after *S. mutans* intravenous injections, bone mineral density of distal femurs was significantly reduced by the bacteria inoculation regardless of which fluorescent reagents were injected subcutaneously. Finally, *S. mutans* inoculation reduced bone-formation-activity indices in both the tetracycline-alizarin double-injected mice and the calcein-alizarin double-injected mice. These results suggested that a one-time injection of tetracycline did not affect bone formation indices in the *S. mutans*-induced bone loss model. Tetracycline could be used for measuring bone formation activity in the same way as non-antibiotic fluorescent reagent such as calcein and alizarin, even in a tetracycline-sensitive bacterium-infected model.

## 1 Introduction

Bone and immune cells share the same microenvironment and interact with each other in the bone marrow, and the bone itself provides a unique harbor for microorganisms (Tsukasaki and Takayanagi, 2019). Recently, a bacteriological challenge was reported to reduce bone mineral density and bone formation activity in cecal ligation and puncture model (Terashima et al., 2016). Although several lines of evidence showed the relationship between bone remodeling and *Porphyromonas gingivalis*, a periodontal disease-related bacteria (Kajiya et al., 2010; Zhang et al., 2014), the relationship between cariogenic bacteria and bone disorders has not yet been widely elucidated. *Streptococcus mutans* (*S. mutans*) is the most common facultative anaerobic gram-positive cocci found in the human oral cavity. It is considered to be the major cariogenic bacterium (Forssten et al., 2010) associated with pyogenic and other infections in various sites, including the mouth, heart, joints, skin, muscle, and central nervous system (Bergy and Holt, 1994). In addition, *S. mutans* have been reported as the most frequently detected species in cardiovascular patients with high detection rates in the heart valve and aneurysm wall specimens of 42.7% and 62.8%, respectively, when compared among oral bacteria (Nakano et al., 2009). It may also be associated with the development of deep cerebral microbleeds, intracerebral hemorrhage, and stroke, with a mechanism linked to chronic inflammation (Tonomura et al., 2016). On the other hand, the bone organ is now known to be interconnected with the nervous, endocrine, and immune system. The latter is closely related to the formation of osteoclasts and osteoblasts and their function (Okamoto et al., 2017). Therefore, it could be an interesting field of work to study the relationship between oral bacteria and bone metabolism, bone formation, and bone resorption. However, there is no established procedure in bone histomorphometric analyses to detect changes in bone formation activity, which can be measured using fluorescent reagents in a bacterium-infected model.

Bone histomorphometry was developed in the 1960s to quantify the balance between bone resorption and bone formation using human iliac biopsy samples administered with fluorescent material (Frost, 1969). Among the bone histomorphometric parameters that quantify bone remodeling using undecalcified sections, the bone formation parameters are considered to be the most reliable indices with minor inter-observer variation (Compston et al., 1986). Usually, tetracycline, a fluorescent reagent, has been long-time used as a clinical bone formation marker that is generally administered to clarify bone formation activity in the morphological analyses using the undecalcified bone specimen (Weinstein et al., 1997; Parfitt, 2002). The measurement of bone formation activity utilizes the fact that a fluorescent reagent is deposited on the calcification front. Various indices of bone formation activity are calculated by measuring the distance between the double fluorescent labels of tetracycline administered at different time points (Dempster et al., 2013). Tetracycline is also applied to animal studies in bone histomorphometric analyses. Since tetracycline is an antibacterial drug, it cannot be ruled out the possibility that the results of experiments using bacteria may be misinterpreted. Over the past several decades, green and red fluorescence have become standard fluorescent reagents in a cell biological study using a confocal laser scanning microscope since a special filter set is necessary to detect tetracycline. Therefore, calcein, green fluorescence, and alizarin, a red fluorescence are preferably used compared to tetracycline, a yellow fluorescence. However, tetracycline is still used in animal studies for measuring bone formation activities, and it has not yet been investigated which fluorescent reagent is suitable for bacterial-infection experiments. Therefore, the purpose of this study was to examine fluorescent reagents suitable for evaluating bone formation activity using infected mice inoculated with *S. mutans* that are considered to be sensitive to tetracycline (Jarvinen et al., 1993). In addition, the effects of *S. mutans* inoculation on bone remodeling were also investigated.

## 2 Materials and Methods

### 2.1 Preparation of *S. mutans* Suspension

A laboratory strain of cariogenic bacteria, *S. mutans* MT8148 strain (Biosafety level 1), was used for this *S. mutans-*infected model. After 16 hrs of preculture, fresh *S. mutans* were cultured with 5 ml of brain heart-infusion broth (BHI; Becton, Dickinson and Company, Sparks, MD, USA) at 37°C under anaerobic conditions using O_2_-absorbing and CO_2_-generating AnaeroPouch (Mitsubishi Gas Chemical Co., Inc., Tokyo, Japan) for 16 hrs, then centrifuged at 3,500 rpm (1330g), 4°C for 10 minutes as described elsewhere (Gyo et al., 2008). The pellet was dissolved with sterile phosphate buffer saline (PBS). The optical density of the bacterial suspension was adjusted by adding up PBS until an optical density of 490 nm (OD_490_) = 1.0 [approximately 3.3 × 10^7^ colony-forming unit (CFU)/ml]. The optical density was recorded using a spectrophotometer (Model 680 Microplate Reader: Bio-Rad, Hercules, CA, USA).

### 2.2 Experimental Design

**Figure 1:**
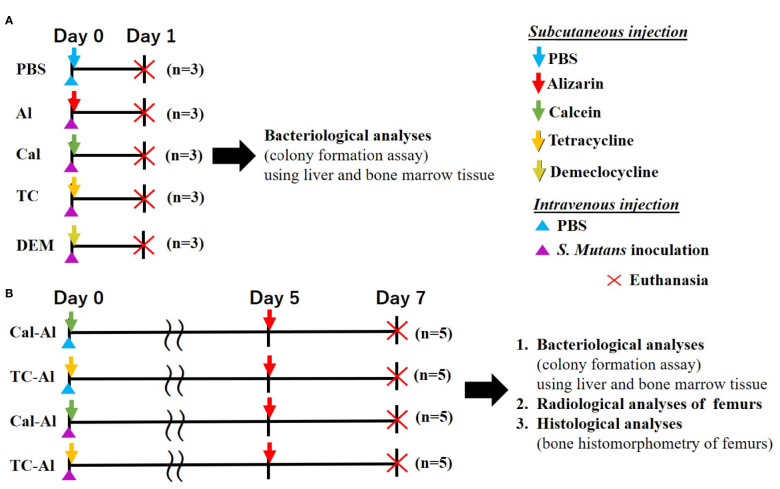
Experimental design. **(A)** One-day experiment comparing the effects of 4 different fluorescent reagents on bacterial colony formation in *S. mutans*-infected mice. Subcutaneous injection of PBS to the intravenously injected mice used as a control **(B)** Seven-day experiment examining the effects of tetracycline versus calcein as a fluorescent reagent bacteriologically, radiologically, and histologically in the *S. mutans-*infected model comparing to the mice received an intravenous injection of PBS.

Thirty-five twelve-week-old male BALB/c mice (Sankyo Labo Service Corporation, INC., Tokyo, Japan) were used. All animals were given a week to adapt to a 12-hour dark/light cycle under constant temperature (21 ± 1°C) with *ad libitum* access to food and water. All mice cages used were sterilized with the sterilized filtered top, and all procedures for feeding and cleaning mice were performed inside a biological safety cabinet. Experimental procedures were approved by the Animal Care and Use Committee of Tokyo Medical and Dental University (Tokyo, Japan, authorization numbers: A2019-216C2). After one week adaptation period, the mice were inoculated with 0.3 ml of above *S. mutans* suspension intravenously into the tail vein of mice sedated with medetomidine hydrochloride (0.75 mg/kg; Domitor^®^, Zenoaq, Fukushima, Japan) subcutaneously injected into the back at a dose of 0.1 ml/10 g mice. The experimental protocol was schematically shown in **Figure 1**; one-day experiment (**Figure 1A**), seven-day experiment (**Figure 1B**).

#### 2.2.1 One-Day Experiment

Fifteen mice were divided into five groups: 1) subcutaneously PBS-injected group immediately after intravenous PBS injection from the tail vein (negative control) (n = 3), 2) alizarin complexone (alizarin; Dojindo, Kumamoto, Japan) -injected group (n = 3), 3) calcein (Sigma-Aldrich) -injected group (n = 3), 4) tetracycline hydrochloride (tetracycline; FUJIFILM Wako Pure Chemical Corp., Osaka, Japan)-injected group (n = 3), and 5) demeclocycline hydrochloride (demeclocycline; Sigma-Aldrich, St. Luis, MO, USA) -injected group (n = 3) (**Figure 1A**). Later four groups received subcutaneous injections of fluorescent reagents using a 27 G needle and a 1 ml syringe (Terumo Corporation, Tokyo, Japan) at a dose of 20 mg/kg immediately after intravenous *S. mutans* inoculation to the tail vein. All mice were sedated and then sacrificed by cervical dislocation 24 hrs after intravenous PBS injection or *S. mutans* inoculation. The mice were immersed in 70% ethyl alcohol for several seconds, and then right femurs and liver were dissected for bacteriological analyses. The comparison between alizarin and PBS subcutaneous injections on the colony formation was shown in the **Supplementary Section**.

#### 2.2.2 Seven-Day Experiment

Twenty mice were divided into four groups: 1) calcein and alizarin double-injected group with the intravenous PBS injection (n = 5), 2) tetracycline and alizarin double-injected group with the intravenous PBS injection (n = 5), 3) calcein and alizarin double-injected group with intravenous *S. mutans* inoculation (n = 5), and 4) tetracycline and alizarin double-injected group with intravenous *S. mutans* inoculation (n = 5) (**Figure 1B**). The first fluorescent reagent was subcutaneously injected immediately after intravenous PBS injection or the *S. mutans* inoculation on day 0. The second fluorescent injection was performed on day 5 after the intravenous PBS injection *S. mutans* inoculation at a dose of 20 mg/kg. On day 7, after the intravenous PBS injection or the intravenous *S. mutans* inoculation, all mice were sedated and sacrificed by cervical dislocation. The right femurs and liver were dissected for bacteriological analyses in the same way as performed in the one-day experiment. The left femurs were also dissected for radiological and histological analyses.

The procedures of dissecting organs and tissues were all performed inside a biological safety cabinet to ensure an aseptic environment and prevent other bacterial contamination.

### 2.3 Bacteriological Analyses of Liver and Bone Marrow Tissue

The dissected liver and right femurs were used for colony measurement. The left lateral lobe of the liver tissue was taken and weighed. The tissue was suspended in 1 ml of PBS and then crushed in PBS with a homogenizer (UP100H, DKSH Japan K.K., Tokyo, Japan). Bone marrow was flushed out from the right femur using a syringe using 1 ml of PBS. The suspension (appx. 50µl each) of liver or bone marrow tissue was seeded on Difco™ Mitis-Salivarius Agar (Becton, Dickinson and Company) plate by using a spiral machine (EDDY JET, Central Science Trading Co., Ltd., Tokyo, Japan). Plates were grown under anaerobic conditions for 48 hrs at 37°C, and colony number was counted in microscopic images according to the manufacturer’s protocol and presented as colony forming units (CFU/ml/mg).

### 2.4 Radiological Analyses

The dissected left femurs were fixed overnight in 3.7% phosphate-buffered formalin solution (pH 7.4) (FUJIFILM Wako Pure Chemical Corp.) at 4°C under constant shaking motion. All samples were then washed with PBS to remove formalin as much as possible. Soft X-ray images were taken with a cabinet X-ray apparatus (type SRO-M50, Sofron, Tokyo, Japan) under the conditions of 28 kV, 4 mA, to preliminarily visualize the calcified tissue and to observe the structural changes of the cancellous region of distal femur diaphysis.

Three-dimensional reconstruction images of femurs were obtained by microfocal computed tomography (μCT) (Scan Xmate-E090; Comscan, Kanagawa, Japan). Microarchitectural changes were then observed using a three-dimensional bone structure reconstruction system (TRI/3D-View; RATOC System Engineering, Tokyo, Japan).

Three-dimensional cross-sectional scanning was performed at 1.5-2.1 mm proximal starting from the distal end of femurs using peripheral quantitative computed tomography (pQCT; XCT Research SA +, Stratec Medizintechnik GmbH, Pforzheim, Germany). The total bone mineral density (BMD), trabecular BMD, and cortical bone thickness at the diaphysis of femurs were obtained from the average of 3 slices of each sample. We used 395 and 690 mg/cm^3^ as thresholds of the trabecular BMD (peel mode 2) and cortical BMD, respectively.

### 2.5 Histological Assessments and Bone Histomorphometry

The dissected left femurs were embedded in Super Cryoembedding Medium compound (SCEM; Section-Lab Co. Ltd, Hiroshima, Japan) and frozen into blocks at −100°C in a freezing machine (UT2000F; Leica Microsystems, Tokyo, Japan). Micro CT images guided the direction of the section cuts of the embedded bone samples. The undecalcified frozen blocks were then cut into slices of 5 µm in thickness using a microtome (CM3050sIV; Leica Microsystems) and the adhesive film (Cryofilm type 2C, Section-Lab, Co., Ltd) for applying the Kawamoto method (Kawamoto, 2003).

The fluorescence-labeled sections were observed under an inverted fluorescence microscope (FSX100; Olympus, Tokyo, Japan), and histomorphometric analyses were performed using an image analyzing system (ImageJ software program, version 1.53i; NIH, Bethesda, MD, USA). All bone histomorphometric measurements were performed according to the standard protocols updated by the American Society of Bone and Mineral Research (Dempster et al., 2013). The region of interest (ROI) for the histomorphometric analyses was set in the secondary spongiosa of the 0.72 mm2 area (1.2 x 0.6 mm) rectangle starting from 0.6 mm proximal femur’s growth plate from the growth plate of the distal femur. Measured parameters were shown as follows; structural parameters: bone volume per tissue volume (BV/TV), trabecular thickness (Tb.Th), trabecular number (Tb.N), and the trabecular separation (Tb.Sp); Bone formation parameters: mineral apposition rate (MAR), mineralizing surface (MS/BS), and bone formation rate (BFR) as bone surface reference. Bone resorption parameter: osteoclast number (N.Oc/BS). Some sections were stained with tartrate-resistant acid phosphatase (TRAP) and counterstained with methyl green to identify osteoclasts. TRAP-positive cells on the bone surface of the trabeculae with two or more nuclei were identified as osteoclasts.

### 2.6 Statistical Analysis

The number of mice of day-seven experiment was determined using the following formula based on the calculated number from the data of trabecular BMD in our pilot study.


n=2(Zα/2+Zβ)2 SD2/Δ2

n, number of specimens, which was expected to get a significant difference in each experimental group

Zα/2 = 1.96: fixed number in the case of Significance = 5%

Zβ =0.84: the fixed number in the case of Power = 80%

SD, Standard deviation

Δ: The difference between the mean value; Av1 and Av2

The difference between the average and standard deviation was determined by the pilot study


$$n=\frac{2*{\left(1.96+0.84\right)}^{2}*{\left(SD\right)}^{2}}{{\left(Av_{1}-Av_{2}\right)}^{2}}$$

Standard deviations and mean difference (SD=14, Av1 - Av2 = 25) were obtained for Total BMD from the results of the pilot study.


$$4.917=\frac{2*{\left(1.96+0.84\right)}^{2}*{\left(14\right)}^{2}}{{\left(25\right)}^{2}}$$

And also, the effect size was calculated by statistical software (G*power 3.1.9.2) after statistical analysis (Faul et al., 2007; Erdfelder et al., 2009). Effect size indicated 1.111 from the result of statistical analysis for ANOVA. This value indicates a strong statistical effect.

Regarding the one-day experiment, the number of mice was determined based on the data of colony counting in our pilot study in the same way as described above. The detail of the calculation for one-day experiment is shown in the **Supplementary Section**.

The distribution of data was analyzed by the Shapiro-Wilk test. The variance of data was analyzed by the Levene test. The comparison among the colony formation experimental groups was analyzed by the Kruskal Wallis test and the Wilcoxon rank-sum test. The comparison of the rest of the results was analyzed by the ANOVA and Tukey HSD test. P-value was performed with p<0.05 as having a significant difference. The data were expressed as mean ± standard deviation. Statistical procedures were performed using SPSS ver.27.0 (IBM, Chicago, USA).

## 3 Results

### 3.1 Tetracycline Injection With the *S. mutans* Inoculation Affected the Colony Formation of Liver and Bone Marrow Tissue on Day One, but Calcein and Alizarin Did Not

We used a bacterial model induced by *S. mutans* to evaluate whether a tetracycline could be an appropriate fluorescent reagent for clarifying the changes of bone formation activity under an infected condition with the tetracycline-sensitive bacteria. Four kinds of fluorescent reagents, which have been often used in bone histomorphometric analyses, were injected in the *S. mutans-*infected model (**Figure 1A**). The dose of fluorescent reagents was the standard dose for bone histomorphometry in a mouse model.

Before starting the experiment shown in **Figure 1A**, we had preliminarily tested the effects of subcutaneous injection of alizarin, one of the representative fluorescent reagents to evaluate bone formation activity, on colony formation of liver and bone marrow tissue on day one after *S. mutans* intravenous inoculation compared to those of subcutaneous PBS injection. Similar numbers of colonies derived from liver and bone marrow tissue appeared in alizarin- and PBS- injected groups after one day of intravenous inoculation of *S. mutans*. Quantitative analyses of the number of colonies derived from the liver and bone marrow tissue showed no significant difference between alizarin- and PBS-injected groups (p>0.05, **Supplementary Figure 1**).

**Figure 2:**
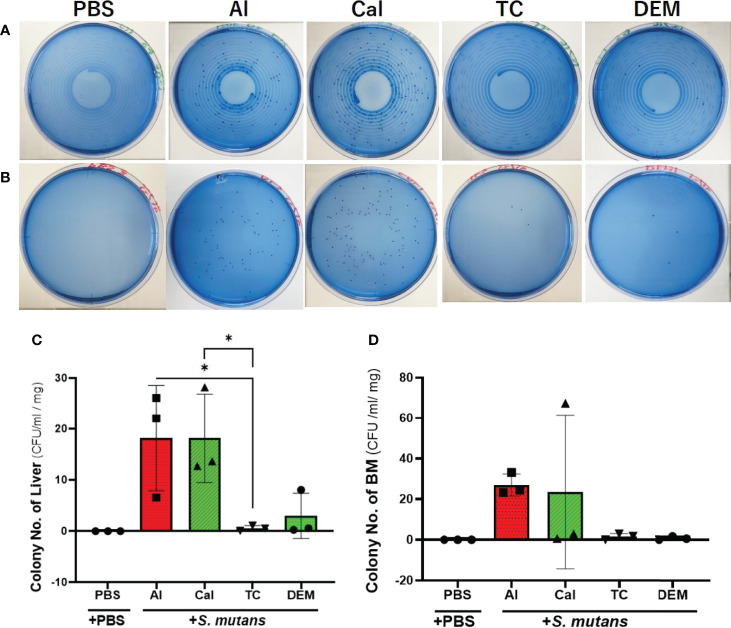
Tetracycline-related fluorescent reagents affected colony formation. Bacteriological analysis of one-day experiment comparing the effects of 4 different fluorescent reagents on the bacterial colony formation in *S. mutans*-infected mice. **(A)** Culture-plate images of liver-derived colonies after one day of *S. mutans* inoculation. **(B)** Culture-plate images of bone marrow (BM)-derived colonies after one day of *S. mutans* inoculation. **(C)** The number of colonies derived from liver. **(D)** The number of colonies derived from bone marrow tissue. PBS, phosphate buffer saline; Al, alizarin; Ca, calcein; TC, tetracycline; DEM, demeclocycline. +PBS, intravenously PBS-injected group; +*S. mutans*, intravenously *S. mutans*-inoculated groups. The variance of data was analyzed by the Levene test. The comparison between groups was analyzed by the Kruskal Wallis test and the Wilcoxon rank-sum test. Values are expressed as the mean ± SD, *p < 0.05.

Tetracycline and demeclocycline injections immediately after the *S. mutans* inoculation lowered the number of colonies derived from liver and bone marrow tissue after one day of *S. mutans* intravenous inoculation (**Figures 2A, B**). Quantitative analyses of the number of colonies from the liver in the tetracycline-injected group showed a significant reduction compared to alizarin-single and calcein-single injected groups (p<0.05, **Figure 2C**). The culture plate of both liver and bone marrow tissues obtained from intravenously PBS-injected mice showed no colony formation on one day after subcutaneous PBS-injections, which was performed immediately after the intravenous *S. mutans* injections. The number of colonies derived from bone marrow similarly appeared as the number of colonies derived from the liver, although there was no significant reduction in the number of colonies due to the tetracycline injection (**Figures 2B, D**).

**Figure 3:**
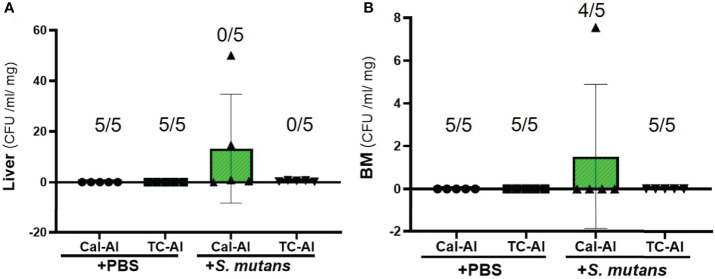
Colony formation from the liver and bone marrow tissue on day 7 after intravenous injection of PBS or *S. mutans* inoculation. Comparison of the number of colonies derived from liver and bone marrow tissue after seven days of *S. mutans* inoculation. **(A)** The number of colonies derived from liver. **(B)** The number of colonies derived from bone marrow tissue. Cal-Al, mice received double injections of calcein and alizarin; TC-Al, mice received double injections of tetracycline and alizarin; +PBS, intravenously PBS-injected groups; *+S. mutans*, intravenously *S. mutans*-inoculated groups. The numerator and denominator numbers inside the bracket represent the numbers of the sample, which shows zero value and the number of all samples, respectively, i.e.: (5/5) means that 5 out of 5 have zero value. The variance of data was analyzed by the Levene test. The comparison between groups was analyzed by the Kruskal Wallis test and the Wilcoxon rank-sum test. Values are expressed as the mean ± SD.

Liver-derived colonies after 7 days of *S. mutans* inoculation with subcutaneous injection of tetracycline on day zero and alizarin on day 5 after *S. mutans* inoculation also reduced compared to the calcein-alizarin double injected group. However, there was no significant difference between tetracycline-alizarin double injected and calcein-alizarin double injected groups (p>0.05, **Figure 3A**). In the observation of colonies derived from bone marrow tissue in mice injected with calcein-alizarin on day 7, one sample out of 5 samples showed colony formation and, while no colony formation was observed in the tetracycline-alizarin double injected group (**Figure 3B**). There was no colony formation appeared in both calcein-alizarin and tetracycline-alizarin injected groups on day 7 after the PBS-intravenously injection (**Figures 3A, B**).

### 3.2 *S. mutans* Inoculation Reduced Bone Mineral Density in the Tetracycline-Injected Group to the Same Extent as in the Calcein-Injected Group

**Figure 4:**
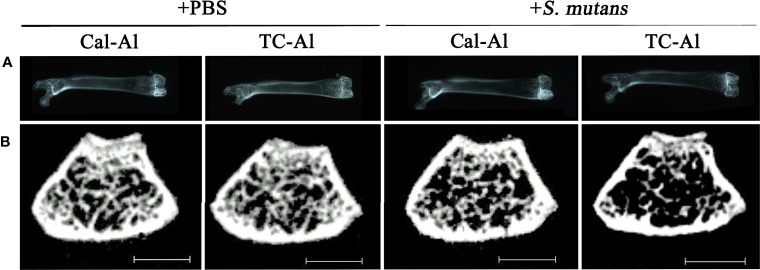
Representative radiological examination of the femur. Radiological analyses of the femurs after seven days of intravenous injections of PBS or *S. mutans* inoculation. **(A)** Representative soft X-ray photographic images of femurs. **(B)** Representative µCT images of a cross-section at the diaphysis of the distal end of the femurs. Cal-Al, mice received double injections of calcein and alizarin; TC-Al, mice received double injections of tetracycline and alizarin; +PBS, intravenously PBS-injected groups; *+S. mutans*, intravenously *S. mutans*-inoculated groups. Scale bar = 1 mm.

Soft X-ray images showed a reduced radio-opacity at the cancellous bone region of distal femurs in the *S. mutans*-inoculated group compared to the PBS control group (**Figure 4A**). Nevertheless, we could not find apparent changes at the cancellous bone region in the tetracycline-alizarin double injected mice compared to the calcein-alizarin double injected mice among the *S. mutans-*inoculation mice (**Figure 4A**). Micro CT images revealed that the *S. mutans*-inoculated groups showed a clear reduction in the cancellous bone region in the distal diaphysis of femurs compared to the PBS-injected control groups (**Figure 4B**). On the other hand, no apparent difference was found between the calcein-alizarin and tetracycline-alizarin double injected groups (**Figure 4B**).

**Figure 5:**
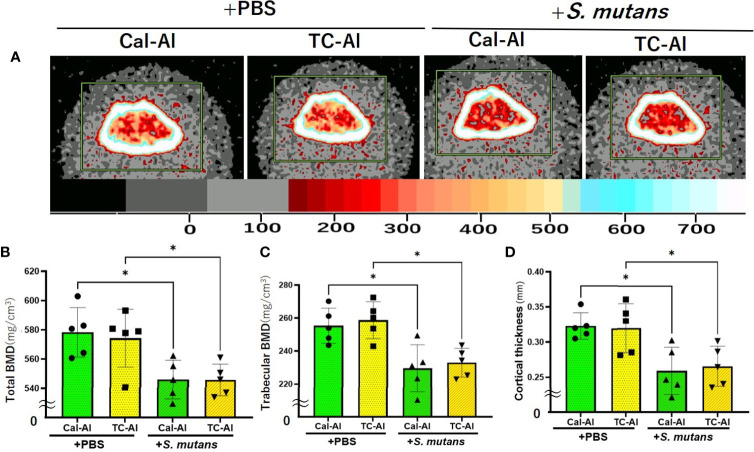
*S. mutans* inoculation reduced bone mineral density. Quantitative analyses of the bone mineral content of the diaphysis of distal femurs after seven days of intravenous injection of PBS or *S. mutans* inoculation. **(A)** Representative pQCT image of the distal femurs for quantitative analyses. The color palette for bone mineral density is shown in the lower bar. **(B)** Total bone mineral density (Total density) of the distal femur; **(C)** Trabecular bone mineral density (Trabecular density) of the distal femur; **(D)** Cortical bone thickness (Cortical thickness) of the distal femur. Cal-Al, mice received double injections of calcein and alizarin; TC-Al, mice received double injections of tetracycline and alizarin; +PBS, intravenously PBS-injected groups; *+S. mutans*, intravenously *S. mutans*-inoculated groups. The variance of data was analyzed by the Levene test. The comparison between groups was analyzed by the ANOVA and Tukey HSD test. Values are expressed as the mean ± SD, *p < 0.05.

pQCT analyses confirmed these observations (**Figures 5A, B**). On day 7, after intravenous injection of PBS or *S. mutans*, total BMD, which includes both cancellous and cortical bone regions at the distal diaphysis of femurs, showed a significant decrease in the *S. mutans*-injected group compared to the PBS-injected group in the calcein-alizarin double injected mice (p<0.05). The trabecular BMD and cortical thickness also showed similar changes to the total BMD (**Figure 5B**). However, no significant difference between calcein-alizarin and tetracycline-alizarin double-injected groups in both PBS and *S. mutans*-injected groups (p>0.05, **Figure 5B**).

### 3.3 A Reduction of Bone Formation Activity in the *S. mutans*-Infected Model Occurred Regardless of the Tetracycline or Calcein Injection

**Figure 6:**
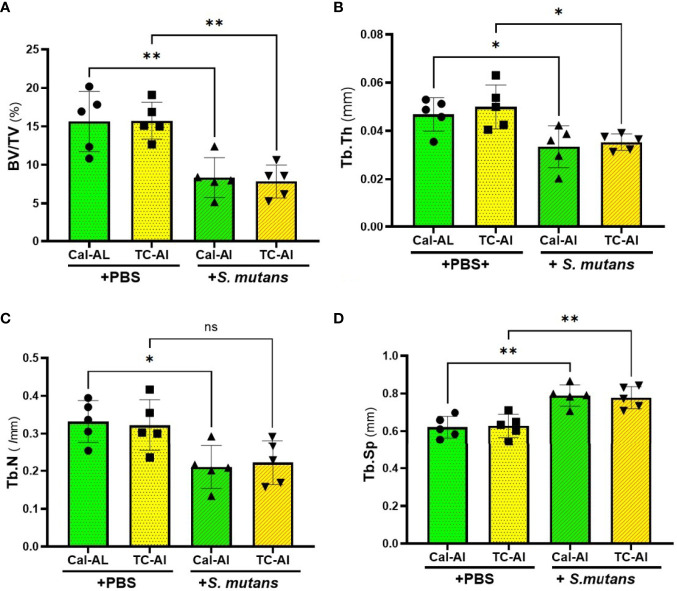
*S. mutans* inoculation decreased structural parameters of bone. Quantitative analyses of the bone structural parameters of the distal femur after seven days of intravenous injection of PBS or *S. mutans* inoculation. **(A)** The ratio of bone volume (BV) to tissue volume (TV), **(B)** Trabecular thickness (Tb.Th), **(C)** Trabecular number (Tb.N), **(D)** Trabecular separation (Tb.Sp). Cal-Al, mice received double injections of calcein and alizarin; TC-Al, mice received double injections of tetracycline and alizarin; +PBS, intravenously PBS-injected groups; *+S. mutans*, intravenously *S. mutans*-inoculated groups. The variance of data was analyzed by the Levene test. The comparison between groups was analyzed by the ANOVA and Tukey HSD test. Values are expressed as the mean ± SD, *p < 0.05, **p < 0.01, ns, no statistically significant difference (p > 0.05).

We used undecalcified thin sections of femurs to clarify the effects of tetracycline fluorescence injection on bone structure, bone formation, and resorption in the *S. mutans*-infected model using the bone histomorphometric analysis. On day 7 after intravenous inoculation of *S. mutans* or PBS injection, bone volume (BV/TV) and trabecular thickness (Tb.Th) were reduced significantly in the *S. mutans*-inoculated groups compared to the PBS-injected groups (p<0.05). However, no significant difference of these parameters was detected between calcein-alizarin and tetracycline-alizarin double injected groups (p>0.05, **Figures 6A, B**). The trabecular number showed a similar tendency to the two structural parameters mentioned above (**Figure 6C**). Trabecular separation showed a significant increase in the *S. mutans*-inoculated groups compared to the PBS-injected groups (p<0.05). However, no significant difference between calcein and tetracycline injections was appeared in the *S. mutans*-inoculated group (p>0.05, **Figure 6D**).

**Figure 7:**
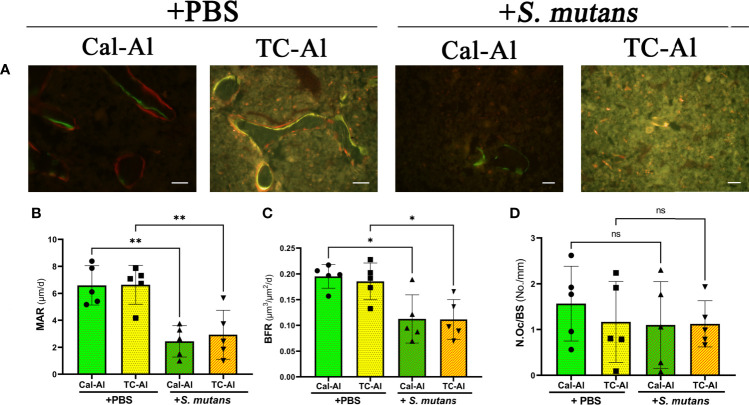
*S. mutans* inoculation decreased structural bone formation parameters. Histological images and bone histomorphometry after seven days of intravenous injection of PBS or *S. mutans* inoculation. **(A)** Representative fluorescence images of undecalcified frozen sections: calcein (green), tetracycline (yellow), and alizarin (red). Scale bar = 50 μm. Quantitative analyses of bone formation activity were performed using standard bone histomorphometric measurement techniques based on the calcein/tetracycline- and alizarin-labeled surface in the ROI, which was described in the *Materials and Methods* section. **(B)** Mineral apposition rate (MAR), **(C)** Bone formation rate (BFR), **(D)** Number of osteoclast per bone surface (N.Oc/BS). Cal-Al, mice received double injections of calcein and alizarin; TC-Al, mice received double injections of tetracycline and alizarin; +PBS, intravenously PBS-injected groups; *+S. mutans*, intravenously *S. mutans*-inoculated groups. The variance of data was analyzed by the Levene test. The comparison between groups was analyzed by the ANOVA and Tukey HSD test. Values are expressed as the mean ± SD, *p < 0.05, **p < 0.01, ns, no significant difference.

Fluorescence images of undecalcified sections revealed that the double fluorescence labeling appeared to be reduced in the *S. mutans*-injected groups compared to the PBS-injected control groups regardless of calcein or tetracycline injections (**Figure 7A**). Quantitative analyses confirmed these observations (**Figures 7B, C**). A significant reduction of MAR, which is calculated from the intra-distance between double fluorescence labeling and shows a function of osteoblasts to form calcified tissues, was detected regardless of calcein or tetracycline injection (p<0.05, **Figure 7B**). The significant reduction of MS/BS, which indicates the active bone formation surface and reflects the number of active osteoblasts, was also detected (p<0.05, **Supplementary Figure 2**). In the same manner, as MAR and MS/BS, BFR, which indicates an amount of bone formed in a day in the ROI of the distal femurs, was significantly reduced compared to the PBS-injected group (p<0.05, **Figure 7C**). There was no significant difference on N.Oc/BS, which indicates a ratio of osteoclasts number per bone surface and a frequency to initiate bone remodeling among examined groups (p>0.05, **Figure 7D** and **Supplementary Figure 3**). As we observed the effects of tetracycline injection on the BMD and the structural parameters, all these bone formation and bone resorption-related parameters did not show any significant difference between the tetracycline-alizarin double injected groups and the calcein-alizarin double injected groups in both the PBS-injected and the *S. mutans*-inoculated groups (p>0.05, **Figures 6** and **7**).

## 4 Discussion

We investigated the feasibility of using tetracycline to measure bone formation activity in a bacteria-infected mice model. The effects of four different fluorescent reagents on the colony formation of liver and bone marrow tissues were compared in the *S. mutans-*infected model. The tetracycline injection reduced the number of colonies derived from the liver after one day of *S. mutans* inoculation (**Figure 2C**). Radiological analyses of the femurs revealed that the *S. mutans* inoculation reduced the total and trabecular BMD in both the calcein-alizarin and the tetracycline-alizarin double injected groups. However, there was no significant difference in these radiological indices between calcein and tetracycline (**Figure 5**). The *S. mutans*-inoculated groups showed an osteopenic phenotype, a significantly lower in structural parameters such as bone volume and trabecular thickness, associated with a significantly higher value in trabecular separation (**Figure 6**) compared to the PBS-injected groups. Then again, no significant difference in these structural parameters was found between the calcein-alizarin and the tetracycline-alizarin double injected groups (**Figure 6**). Finally, fluorescence images of undecalcified sections revealed that bone formation activity was significantly lower in the *S. mutans* groups compared to the PBS control groups. It had no significant difference between the calcein- and tetracycline-injected groups (**Figures 7A–C**). Bone resorption parameter showed similar values regardless of calcein- or tetracycline-injection (**Figure 7D**). These results suggest that one-time injection of tetracycline to measure bone formation indices did not affect bone formation and resorption in the bacterial model induced by tetracycline-sensitive bacteria even though it could reduce the live bacteria derived from the liver of the infected mice (**Figure 2C**).

As described above, a one-time injection of tetracycline did not ameliorate the reduction of BMD and bone formation caused by *S. mutans*, despite its ability to kill bacteria shown in **Figure 2**, suggesting that tetracycline might not interfere with the inhibitory effects of *S. mutans* on bone formation in this study. We might explain this discrepancy from the pharmacological point of view. When the estimated blood concentration of tetracycline was calculated from the usual fluoresce dose for bone histomorphometry, it would be around 200 µg/ml if the injection was performed intravenously. In this study, we performed a subcutaneous injection, which was thought to deliver less than one-third (66.7 µg/ml) of the maximum blood concentration in case of intravenous injection. The estimated concentration was still considerably higher than the minimum inhibitory concentration (MIC) of tetracycline for *S. mutans* (Jarvinen et al., 1993). This estimated concentration of tetracycline might explain the inhibitory effects on colony formation of the tissues on day one (**Figure 2**). However, antibiotics are usually administered periodically to maintain the blood concentration above MIC, having antibacterial effects *in vivo*. In this study, we injected tetracycline only once simultaneously with the *S. mutans* inoculation and did not inject it periodically. This tetracycline-injection schedule to measure the bone formation activity might not affect bone remodeling because tetracycline injection could not recover the *S. mutans*-induced decrease of BMD and bone formation (**Figures 5** and **7**).

On the other hand, there was a possibility that tetracycline might directly affect osteoblasts and osteoclasts since tetracycline is reported to promote osteoblast differentiation (Sasaki et al., 1991) and inhibit osteoclastogenesis *via* MMP-9 inhibition (Kim et al., 2019). This study did not show any tetracycline-induced increase of bone formation and decreased osteoclast number regardless of the PBS or the *S. mutans* injection. Therefore, from our results, we conclude that tetracycline could be an appropriate fluorescent reagent for investigating bone formation activity, and it could be used even in a tetracycline-sensitive bacterium-induced model. From the histomorphometric point of view, tetracycline could also be explained as an appropriate fluorescence to clarify bone formation activity. The main concern when tetracycline is used in the younger age of mice compared to our study is the discoloration of the teeth in the dental field of work, and stronger effects of tetracycline as antibiotics (Cohlan and Bevelander, 1963). However, we assumed the effects of tetracycline in the younger mice on bone remodeling could not be discussed since the remodeling activity in the growing stage appears much lower than that in the adult stage and the higher rate of bone modeling at the growing stage would definitely affect the bone remodeling indices, which could not lead the evidence to show the effects of the antibiotic, tetracycline by using younger mice than 12 week-old. Usually, two kinds of fluorescent reagents are administered at least several days apart to measure bone formation of the bone specimen. If only one type of reagent is used and injected twice, it would sometimes be difficult to distinguish the two-fluorescent labeling along the bone surface to measure MAR since the clarification of the order of the fluorescent injections and the direction of forming bone is necessary to measure MAR. In our study, tetracycline was administered only once on the day of *S. mutans* injection, indicating that the effects of the antibiotic are very limited. Therefore, tetracycline injection could scarcely matter so long as it is injected only once during the experimental period for the histomorphometric analyses.

Several concerns remain in this study to clarify the effects of the *S. mutans* injection on the colony formation of liver and bone marrow tissues. First, we did not perform any tests to confirm whether the colonies formed were *S. mutans*. However, the colonies we observed could be mainly derived from *S. mutans* since the PBS-injected control group under the condition of no *S. mutans* inoculation did not show any colony formation (**Figures 2** and **3**). Of course, it is necessary to perform the polymerase chain reaction (PCR) following the sequence reading of the PCR products or to use the modified *S. mutans*, such as *S. mutans* inserted a luciferase gene cassette to determine whether the colony we observed was *S. mutans*. Second, we could not clarify whether the bacteria remaining in the liver on day one would grow again until the day of sacrifice. As shown in **Figure 3A**, the colony formation derived from the liver was observed even though the numbers of the colonies were limited on the plate from both the calcein-alizarin double injected and the tetracycline-alizarin double injected groups on day seven after *S. mutans* inoculation. Still, we could not determine whether these colonies on day seven were also derived from *S. mutans* for the same reason described above. Therefore, time-course studies should be examined at shorter time intervals, like three or five days other than performing the PCR analyses, and the recovery of *S. mutans* might be detected. Third, we could not explain why the colonies derived from bone marrow tissue varied very much, and the number of the colonies was less compared to the liver tissue (**Figures 2** and **3**). In this study, bone marrow tissue was examined as it is the origin of bone cells and subsequently reflects the bacterial cell’s effect on bone metabolism. The liver tissue was selected for clarifying the growth of the bacteria as it is known to work as a lymphoid tissue in response to the bacterial attack, and it plays a major role in rapidly clearing bacteria from the bloodstream (Gregory et al., 1996). Further studies are necessary to clarify the immune response and/or the ability of the tissue on the clearance of bacteria, comparing bone marrow tissue with the liver.

In this study, we found a significant reduction of the BMD of femurs on day seven after the intravenous injection of 1×10^7^ CFU *S. mutans*. Although the amount of the bacteria injected was determined according to a recent study (Naka et al., 2016), still, we do not know how much amount of *S. mutans* could reduce the BMD of femurs and whether the amount of the bacteria we used in this study reflects a low-hygiene circumstance of the oral cavity in humans. Furthermore, there is currently no data available that accurately represents the average amount of *S. mutans*, which enters the bloodstream after a dental procedure or daily tooth brushing. Continuous investigation is necessary to establish a new mice model to clarify the relationship between oral bacteria and systemic diseases. On the other hand, the reduction of the BMD induced in the other bacterial model was reported (Terashima et al., 2016). Their results support our present data, showing that the mechanism of the BMD reduction in their bacterial model is explained by reducing bone formation activity and no significant changes of bone resorption index (**Figure 7**). Since MS/BS reflects the active bone-forming surface per bone surface, the values of MS/BS are closely related to the osteoblast number or osteoblast surface per bone surface. As we showed the reduced MS/BS compared to the PBS control group (**Supplementary Figure 2**), osteoblast number might be reduced in our bacterial model. Terashima et al. also proposed the importance of osteoblasts, which produced interleukin 7 to maintain common lymphocyte precursors in the bone marrow tissue. Since these lymphocyte precursors are responsible for producing both T cells and B cells, the reduced osteoblasts might lead to immunosuppression in our *S. mutans-*infected model by reducing the number of lymphocytes. Regarding the relationship between *S. mutans* and bone resorption, we did not observe any significant increase in bone resorption index, as shown in **Figure 7D** although it was reported that Mutan, a protein extract from *S. mutans*, induced osteoclast differentiation and promoted alveolar bone loss (Kwon et al., 2016). Additional studies are necessary to clarify whether the *S. mutans*-induced immune response causes an increase of osteoclasts or *S. mutans* directly affect osteoclast differentiation.

## 5 Conclusions

Tetracycline was shown to be an appropriate fluorescent reagent to measure a bone formation activity even in the tetracycline-sensitive bacterium-induced animal model when it was injected once during an experimental period. In addition, intravenous inoculation of *S. mutans* led to the reduction of bone volume, probably by reducing the bone formation activity without changing osteoclastogenesis.

## Data Availability Statement

The raw data supporting the conclusions of this article will be made available by the authors, without undue reservation.

## Ethics Statement

The animal study was reviewed and approved by The Animal Care and Use Committee of Tokyo Medical and Dental University (Tokyo, Japan, authorization numbers: A2019-216C2.

## Author Contributions

YH performed all experiments and drafted the manuscript. SK helped in all experiments, including radiological analyses and statistics of the data, and revised the manuscript. They have contributed equally to this work and share the first authorship. MK contributed to all animal experiments especially helping intravenous injections to the tail vein, taking soft X-ray images, and making undecalcified sections. MI performed the statistics required for this paper. MO provided suggestions and significantly revised and refined the manuscript. KM supervised the bacterial culture section. FR contributed to the experimental part, analyzed data, and wrote the manuscript. KA received a grant for this project, conceived the idea, reviewed the drafts, supervised the writing process, and edited this manuscript. All authors contributed to the article and approved the submitted version.

## Funding

This work was supported in part by the Company of Biologists’ grants-Travelling Fellowship - 232 Number: JCSTF1908265 to FR; Nakatani Foundation for Advancement of Measuring Technologies 233 in Biomedical Engineering, Technical Exchange Grant (study in Japan) to KA; and the Japan Society for the 234 Promotion of Science (18K19637 and 19H01068 to KA).

## Conflict of Interest

The authors declare that the research was conducted in the absence of any commercial or financial relationships that could be construed as a potential conflict of interest.

## Publisher’s Note

All claims expressed in this article are solely those of the authors and do not necessarily represent those of their affiliated organizations, or those of the publisher, the editors and the reviewers. Any product that may be evaluated in this article, or claim that may be made by its manufacturer, is not guaranteed or endorsed by the publisher.
