# Supplementary material for: Tetracycline, an Appropriate Reagent for Measuring Bone-Formation Activity in the Murine Model of the Streptococcus mutans-Induced Bone Loss
Source: Front Cell Infect Microbiol. 2021 Sep 13;11:714366. doi: 10.3389/fcimb.2021.714366 (PMC8473704; doi:10.3389/fcimb.2021.714366)
Supplement: Supplementary file 1 [file DataSheet_1.pdf]

Supplementary Material

1 Supplementary Data

1.1 Materials and Methods

Six mice were divided into 2 groups: 1) alizarin complexone-injected group (n = 3), 2) PBS-injected group (n = 3). Both groups received subcutaneous injections of fluorescent reagents (using 1 ml syringe at a dose of 20 mg/kg) or PBS using a 27 G needle immediately after the intravenous *S. mutans* inoculation (approximately 1×10^7 colony-forming unit (CFU)). All mice were sedated and then sacrificed by cervical dislocation 24 hrs after intravenous *S. mutans* inoculation. The mice were immersed in 70% ethyl alcohol for several seconds, and then right femurs and liver were dissected for bacteriological analyses. All procedures were performed inside a biological safety cabinet to ensure an aseptic environment and to prevent other bacterial contamination.

The histological and other procedures leading the bone histomorphometric index, mineralizing surface per bone surface (MS/BS), were described in the main text. Some sections were stained with tartrate-resistant acid phosphatase (TRAP) and counterstained with methyl green to identify osteoclasts.

Statistical analysis

Regarding the one-day experiment, the number of mice was determined using the following formula based on the data of colony counting in our pilot study

$n=2(Z_{\alpha/2}+Z_{\beta})^2\ SD^2 / \Delta^2$
n: number of specimens, which was expected to get a significant difference in each experimental group
$Z_{\alpha/2}$ =1.96: fixed number in the case of Significance = 5%
Z_{β} =0.84: the fixed number in the case of Power = 80 %
SD: Standard deviation,
Δ: The difference between the mean value; Av_1 and Av_2
The difference between the average and standard deviation was determined by the pilot study

$$n = \frac{2 * (1.96 + 0.84)^2 * (SD)^2}{(Av_1 - Av_2)^2}$$

The number of mice for a count of Colony No. of Liver was decided by the following formula. Standard deviations and mean differences (SD=8, Av_1 - Av_2=20) were obtained from our pilot study.

$$2.509 = \frac{2 * (1.96 + 0.84)^2 * (8)^2}{(20)^2}$$

The number of mice of the seven-day experiment was determined based on our pilot study. Details were shown in the main text.

The distribution of data was analyzed by the Shapiro-Wilk test. The variance of data was analyzed by the Levene test. The comparison between the colony formation experimental groups was analyzed by the Wilcoxon rank-sum test. The comparison of MS/BS was analyzed by the ANOVA and Tukey HSD test. P-value was performed with $p<0.05$ as having a significant difference. The data were expressed as mean ± standard deviation. Statistical procedures were performed using SPSS ver.27.0 (IBM, Chicago, USA).

1.2 Results

The results on colony formation were also described in the main text. Alizarin and PBS injections showed almost the same number of colonies derived from both liver and bone marrow tissues after one day of *S. mutans* intravenous inoculation. The number of colonies of the liver and bone marrow tissue showed no significant difference between alizarin- and PBS-injected groups (Supplementary Fig. 1).

The results on MS/BS were also described in the main text. Briefly, the significant reduction of MS/BS was detected regardless of calcein or tetracycline injection ($p<0.05$, Supplementary Fig. 2). On the other hand, there was no apparent difference in the appearance of TRAP-positive multinucleated cells among examined groups although bone area in the *S. mutans*-injected groups seemed to be less compared to PBS-injected controls (Supplementary Fig. 3).

2 Supplementary Figures

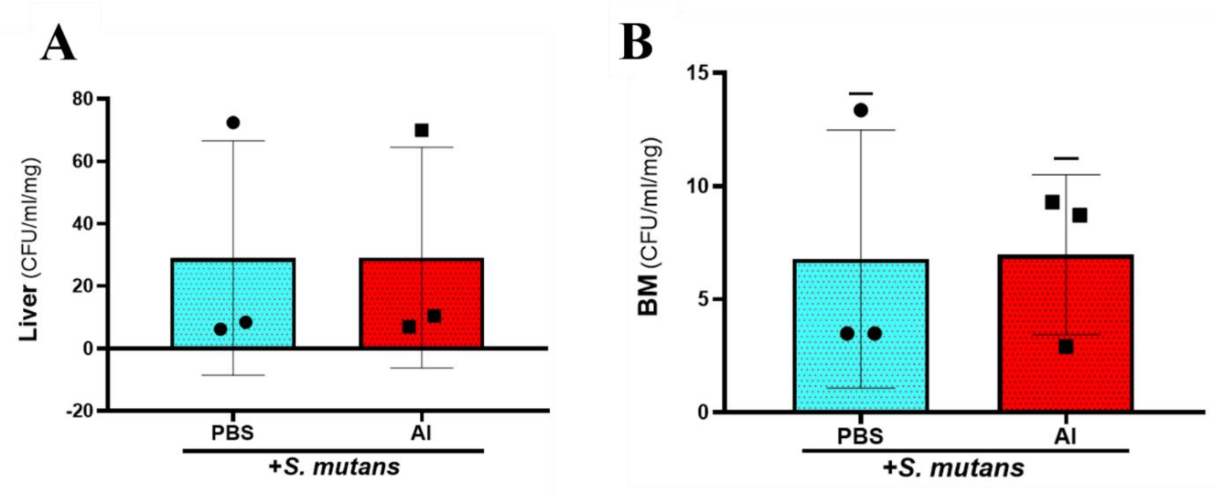

Supplementary Figure 1. The effects of subcutaneous alizarin injection on the colony formation from liver and bone marrow tissue on day 1 after intravenous injection of *S. mutans* inoculation. Representative fluorescent reagent alizarin did not affect colony formation of liver and bone marrow tissue obtained from *S. mutans*-infected mice on day 1 after intravenous inoculation of the bacteria. (**A**) The number of colonies derived from liver. (**B**) The number of colonies derived from bone marrow tissue after one day of *S. mutans* inoculation (BM). **PBS:** phosphate buffer saline, **Al**: Alizarin. **+PBS**: intravenously PBS-injected groups, **+*S. mutans***: intravenously *S. mutans*-inoculated groups. The variance of data was analyzed by the Levene test. The comparison between groups was analyzed by the one sample t-test Values are expressed as the mean ± SD.

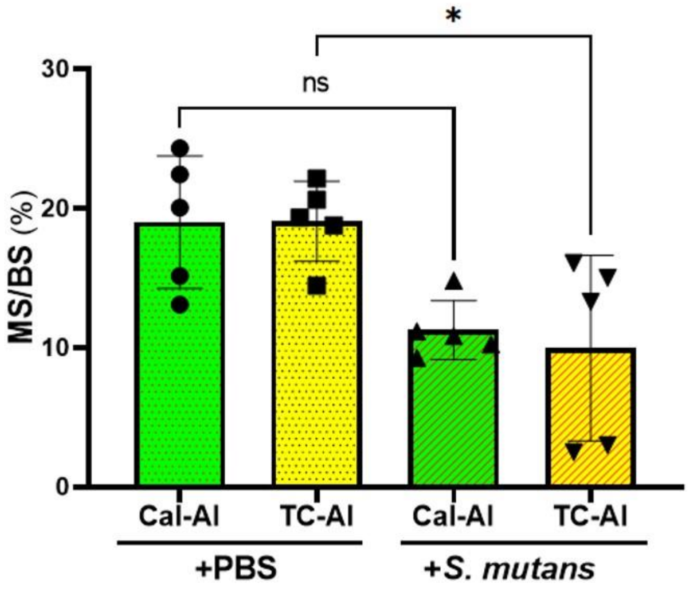

Supplementary Figure 2. ***S. mutans*** **inoculation decreased a structural bone formation parameter.** Mineralizing surface per bone surface (MS/BS) after seven days of intravenous injection of PBS or *S. mutans* inoculation. Quantitative analyses of bone formation activity were performed using standard bone histomorphometric measurement techniques based on the calcein/tetracycline- and alizarin-labeled surface in the ROI described in the main text (Materials and Methods section). Mineralizing surface per bone surface (**MS/BS**). **Cal-Al**: mice received double injections of calcein and alizarin. **TC-Al:** mice received double injections of tetracycline and alizarin. **+PBS**: intravenously PBS-injected groups, **+*S. mutans***: intravenously *S. mutans*-inoculated group. The variance of data was analyzed by the Levene test. The comparison between groups was analyzed by the ANOVA and Tukey HSD test. Values are expressed as the mean ± SD, *p < 0.05, ns: no statistically significant difference (p>0.05).

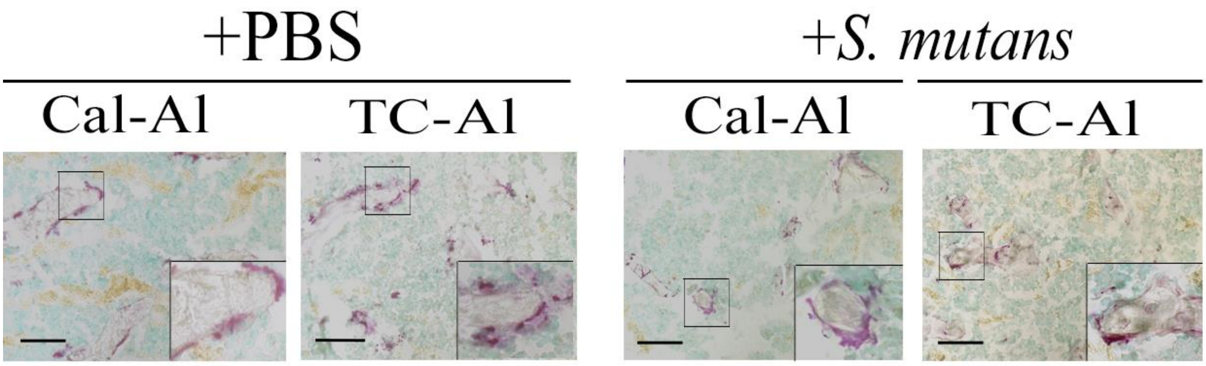

Supplementary Figure 3. Similar appearance of TRAP-positive cells among all experimental groups. Histological images after seven days of intravenous PBS injection or *S. mutans* inoculation. **Cal-Al**: mice received double injections of calcein and alizarin. **TC-Al:** mice received double injections of tetracycline and alizarin. **+PBS**: intravenously PBS-injected groups, **+*S. mutans***: intravenously *S. mutans*-inoculated groups. TRAP-stained undecalcified sections counterstained with methyl green stain. Scale bar = 0.1 mm.
